# Detecting Screams From Home Audio Recordings to Identify Tantrums: Exploratory Study Using Transfer Machine Learning

**DOI:** 10.2196/18279

**Published:** 2020-06-16

**Authors:** Rebecca O'Donovan, Emre Sezgin, Sven Bambach, Eric Butter, Simon Lin

**Affiliations:** 1 The Abigail Wexner Research Institute Nationwide Children's Hospital Columbus, OH United States; 2 Department of Psychology Nationwide Children's Hospital Columbus, OH United States

**Keywords:** machine learning, scream detection, audio event detection, tantrum identification, autism, behavioral disorder, data-driven approach

## Abstract

**Background:**

Qualitative self- or parent-reports used in assessing children’s behavioral disorders are often inconvenient to collect and can be misleading due to missing information, rater biases, and limited validity. A data-driven approach to quantify behavioral disorders could alleviate these concerns. This study proposes a machine learning approach to identify screams in voice recordings that avoids the need to gather large amounts of clinical data for model training.

**Objective:**

The goal of this study is to evaluate if a machine learning model trained only on publicly available audio data sets could be used to detect screaming sounds in audio streams captured in an at-home setting.

**Methods:**

Two sets of audio samples were prepared to evaluate the model: a subset of the publicly available AudioSet data set and a set of audio data extracted from the TV show Supernanny, which was chosen for its similarity to clinical data. Scream events were manually annotated for the Supernanny data, and existing annotations were refined for the AudioSet data. Audio feature extraction was performed with a convolutional neural network pretrained on AudioSet. A gradient-boosted tree model was trained and cross-validated for scream classification on the AudioSet data and then validated independently on the Supernanny audio.

**Results:**

On the held-out AudioSet clips, the model achieved a receiver operating characteristic (ROC)–area under the curve (AUC) of 0.86. The same model applied to three full episodes of Supernanny audio achieved an ROC-AUC of 0.95 and an average precision (positive predictive value) of 42% despite screams only making up 1.3% (n=92/7166 seconds) of the total run time.

**Conclusions:**

These results suggest that a scream-detection model trained with publicly available data could be valuable for monitoring clinical recordings and identifying tantrums as opposed to depending on collecting costly privacy-protected clinical data for model training.

## Introduction

One of the challenges in studying and diagnosing children with (potential) behavioral disorders is the relative unreliability of the information available. Children with autism and other social or disruptive behavior disorders often report their own behavior inaccurately in interviews and on self-report questionnaires [1,2]. Parental reports of behavior, although found to be more accurate than child self-reports [1], can be influenced by parents’ own adjustment [3] and their desire to either exaggerate or diminish familial problems [4]. Parents’ reports can also be affected by poor recall of events and confusion about survey or interview questions. A quantitative, data-driven approach for evaluating behavioral problems would alleviate the need to rely on these potentially inaccurate reports.

With this goal in mind, our study focuses on detecting human screaming within continuous audio recording from inside a family’s home. Real-life audio data streams collected from homes have already proven useful in the diagnosis and treatment of behavioral problems associated with autism [5-7]. Segmenting home audio to capture screams, with some postprocessing, could eventually allow researchers to use the scream as a proxy for temper tantrums or negative interactions between family members [8]. Analyzing these interactions would allow clinicians to assess family relationships in a more objective manner than direct self-report [9], but identifying the segments manually would be tedious and time intensive.

Although much research has been done on audio event classification, this study investigates the ability of a scream-detection model to make useful predictions for audio outside of the training data, which to our knowledge has not yet been addressed in previous work. As such, this work can be considered a proof-of-concept study, demonstrating that a model trained exclusively with publicly available audio data can be used to detect screams in clinical data that would otherwise be costly to collect.

## Methods

The ideal way to train a machine learning model for clinical use would be to obtain large amounts of clinical data and hold out certain examples to use for testing and validation. However, relevant clinical data (eg, home audio recordings of families with children) can often be hard to obtain. We trained the classifier on a publicly available audio database, then approximated clinical data for validation using episodes of the TV show Supernanny.

Our training data is based on portions of the AudioSet data set, “an expanding ontology of 632 audio event classes and a collection of 2,084,320 human-labeled 10-second sound clips drawn from YouTube videos” [10]. Each AudioSet clip has been watched by a human annotator and labeled with any number of the 632 sound classes as clips can contain consecutive or overlapping audio events. For our training set, we compiled all clips annotated with the label “Scream.” AudioSet defines this sound as, “A sharp, high-pitched human vocalization; often an instinctive action indicating fear, pain, surprise, joy, anger, etc. Verbal content is absent or overwhelmed, unlike Shout and Yell” [10], and does not make distinctions between the emotions producing the scream sound.

Additionally, validation of the AudioSet annotations was performed in two ways. First, several of the “Scream”-labeled clips did not contain screaming, or it was nearly indistinguishable, so these annotations were corrected. Second, annotating the on- and offset of a scream (on 1-second intervals) within each clip was necessary because most audio clips did not contain a full 10 seconds of their labeled sound. The resulting data set was composed of 3764 seconds of screaming audio tracks (and the same amount of randomly sampled nonscream audio) from 1158 different AudioSet clips. Additional data augmentation was done by duplicating each sound sample and adding white noise at a 20 dB signal-to-noise ratio (SNR), resulting in a final balanced set of ~15,000 seconds of audio.

**Figure 1:**
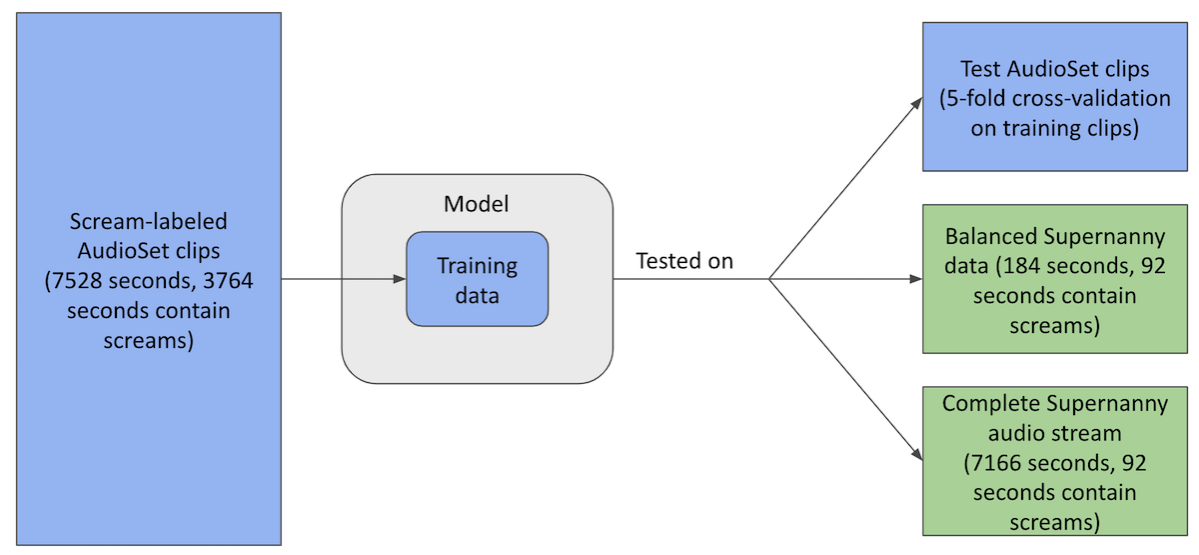
Study design. The model was built using the AudioSet clips by standard 5-fold cross-validation. The model was then tested on Supernanny data for transferability.

The target use case for the model is home audio recordings of families with children, which would likely contain normal conversation, arguing, screaming, and crying as well as silence, background noise, and household noises. In the absence of available clinical recordings of this type, episodes of the TV show Supernanny were used as stand-ins. Each episode focuses on a family in which the parents are struggling to control the behavior of their children. The “Supernanny” (Jo Frost) observes the family, then teaches the parents alternative strategies for discipline and parenting [11]. Supernanny contains several elements that would also be present in clinical data: genuine arguments, children screaming and crying, normal family conversations, and ambient noise. It also contains positive (scream) and negative (nonscream) data points from the same environment (the family’s home) as clinical data would. Three Supernanny episodes were selected and annotated with the same process as the AudioSet clips; they were watched by a human annotator, and every second of audio data was classified as either scream or nonscream. Figure 1 depicts the role of Supernanny audio in relation to other data used in the study.

Two approaches were considered for audio feature extraction. The first was the OpenSMILE (audEERING GmbH) feature extraction software [12], which can extract a range of feature sets using different configuration files. For example, mel-frequency cepstral coefficients (MFCCs) for speech recognition can be extracted, as well as feature sets tuned specifically for emotion recognition [12]. The second approach considered was Google’s open-source “VGGish” convolutional neural network (CNN) for feature extraction [13]. Google’s VGGish network was chosen for two reasons: first, the research behind its feature extraction is more current, and second, it was used in similar recent classification tasks [14]. Functionally, the network analyzes audio and produces a 128-dimensional feature vector extracted by transforming the spectrogram (frequency information) of the audio sample. Since it processes audio data in 960 ms segments, we classified training and test data in 1-second intervals for compatibility.

The original CNN was trained on audio from YouTube-8M, a database of 8 million YouTube videos with over 30,000 class labels, so the features it produces are suitable for audio event classification [13]. We did not fine-tune the VGGish network and used it only as a feature extractor. For classification, Scikit-learn’s gradient boosting classifier was selected for practical reasons (speed of training and ease of use) [15]. This type of classifier iteratively creates an ensemble of simple decision trees such that each new tree tries to correct the previous trees’ biases.

## Results

### Quantitative Evaluation

**Figure 2:**
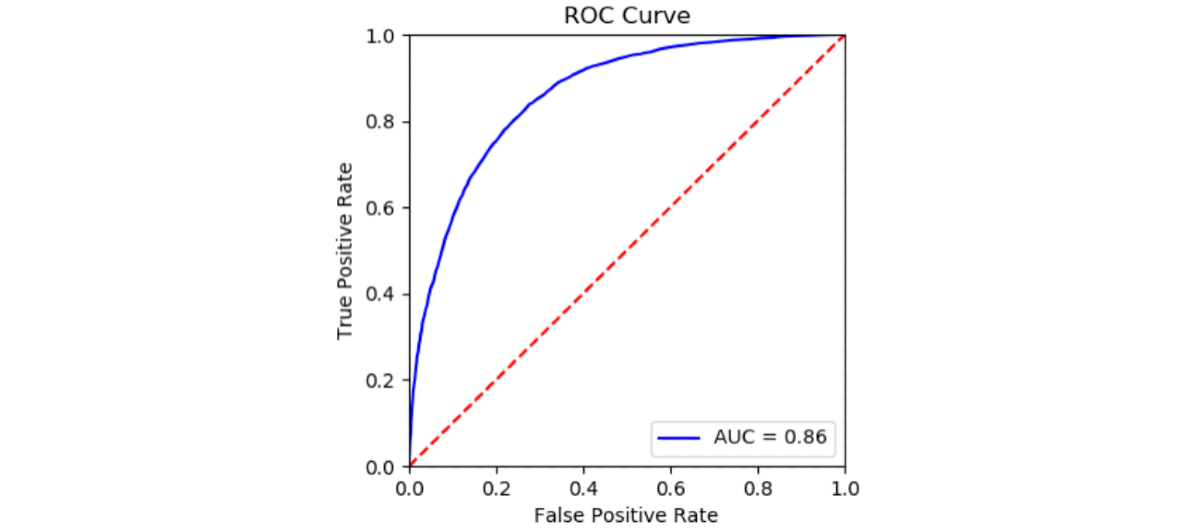
Scream classification performance on the AudioSet data. AUC: area under the curve; ROC: receiver operating characteristic.

The classifier was trained and cross-validated on the class-balanced scream data set of AudioSet clips. The receiver operating characteristic (ROC) curve from a 5-fold cross-validation is shown in Figure 2. The area under the curve (AUC) is 0.86, indicating that our model successfully learned to classify screams using the out-of-the-box VGGish neural network as a feature extractor.

**Figure 3:**
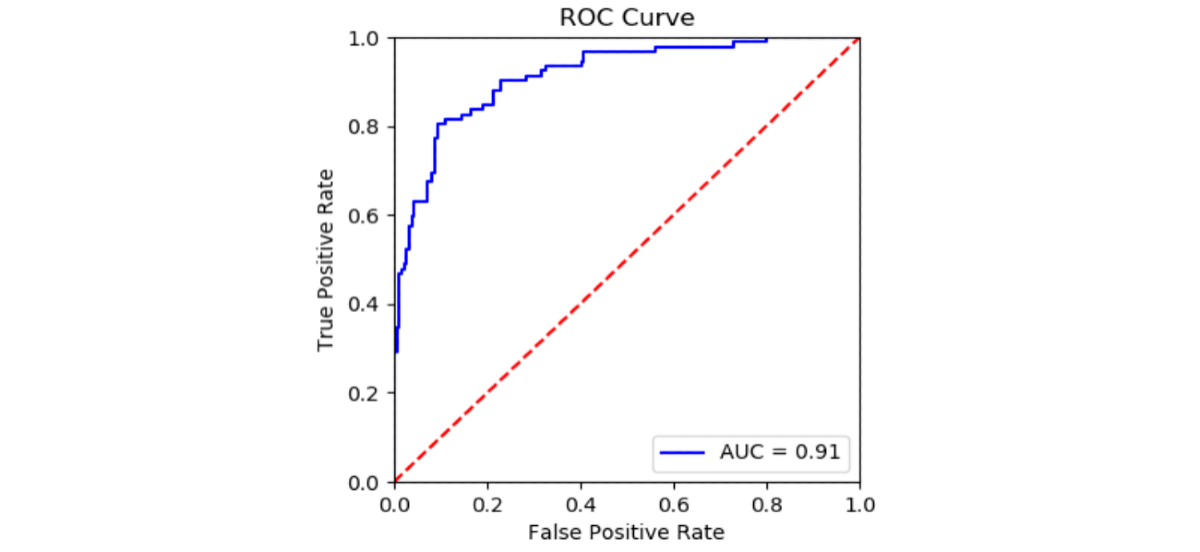
Results on balanced Supernanny data. AUC: area under the curve; ROC: receiver operating characteristic.

In the Supernanny data set, screams occur relatively rarely: only 92 seconds, or 1.3%, of the audio contained screaming out of the full 2 hours (7166 seconds) of annotated audio. Thus, two test sets of Supernanny audio were prepared. The first was artificially balanced by taking every positively marked second, then randomly sampling the same amount of negatively marked data points throughout the audio. The ROC curve for this test set is shown in Figure 3, achieving an AUC of 0.91. This result demonstrates that our model successfully transfers to correctly classifying screams in Supernanny episodes without the need of additional training.

Additionally, we tested the model on the entire audio stream of all three Supernanny episodes. For this evaluation, we incorporated a 1-second margin of error for false positives. For example, if the ground truth annotations for an audio sample contained a scream at second 6 but the model predicted the scream at second 7, the model prediction for the scream would be counted as correct. There were two motivating factors for this approach. First, it accounts for small errors and imprecisions in the ground truth annotations (ie, if an annotator marked the onset of a scream slightly earlier or later than it actually occurred). Second, our model outputs predictions for 960 ms segments of audio, but our ground truth annotations are on a 1-second grid. To test the model’s predictions, we match each (960 ms) prediction to the ground truth second it most overlaps with. Using a 1-second margin of error helps to not penalize the model for misalignment with our ground truth.

**Figure 4:**
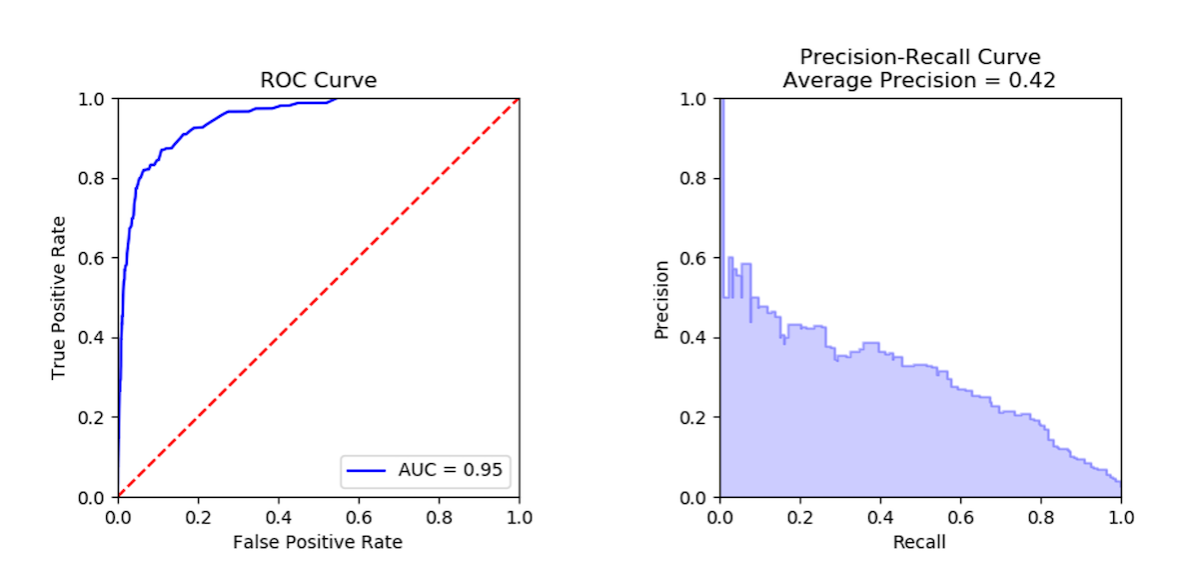
Results on all Supernanny data. AUC: area under the curve; ROC: receiver operating characteristic.

The ROC curve is shown in Figure 4 (left). The slight increase in AUC compared to Figure 3 is likely caused by our 1-second margin of error. Because ROC curves can be misleading in evaluating classifiers for rare events [16], we also provide the precision-recall curve in Figure 4 (right), indicating an average precision (positive predictive value) of 0.42.

### Qualitative Evaluation

To evaluate the types of errors that our model made, we manually examined its predictions on a single episode of Supernanny. We found that many of the false negatives (actual screams that the model missed) were higher-frequency screams by children. We found that of the 9 false positives (seconds the model marked as screams but were not marked as screams by the human annotator) in the episode, 3 seemed to result from very short (<1 second) screams that we chose not to mark as positives during annotation, and 2 occurred on scream-like sounds like loud laughter and crying.

## Discussion

### Principal Results

The most significant finding of this study is that a scream detection model trained only on publicly available data sets had success making predictions on an independent data set containing child screams. Although we have not shown the ability to generalize directly to clinical data, we have shown generalizability from a public database to outside recordings containing home audio characteristics. One of the most significant quantitative results is the average precision value of 0.42 when evaluating the entire Supernanny audio stream, in which screams have a frequency of only 1.3%. A relatively high precision is important for a practical application of the model, especially as we expect scream events in real continuous home audio recordings to be rare as well.

Several characteristics of the proxy test data may bode well for the model’s ability to classify clinical data. One of the most significant differences between the TV show and presumed clinical data is the presence of high-volume, overlapping sound events like voiceover narration and background music in the TV show. Intuitively, loud overlapping audio events would increase the difficulty of classifying the scream event. Sounds are harder to identify when paired with other louder sounds, and model performance on similar data has been shown to suffer as SNR decreases [17]. Thus, it is possible that the classifier would actually perform better on less noisy clinical recordings than it has on the Supernanny audio streams.

Generalizability from public to clinical data has value because it could allow classifiers for clinical recordings to train on public databases. Including patient data in model training sets causes significant risks and inconveniences; for example, if clinical data were directly obtained or reverse-engineered from the trained classifier, patients’ home recordings would be vulnerable to third parties. In addition, consent procedures for inclusion of clinical recordings in training data would be extensive and time-consuming given the number of samples necessary for training in most cases.

### Limitations

This study does not include any clinical data and instead relies on a proxy test data set. Thus, differences between the proxy data (the TV show Supernanny) and the “real-world” data we anticipate using (audio recordings from family homes) constitute limitations. Supernanny contains several audio elements that would not appear in clinical recordings (eg, pervasive background music, voiceover narration, scene cuts). In addition, home audio recordings would include long stretches of silence and everyday conversation that the Supernanny episodes do not include. It is possible that the AudioSet training data has more similarities to Supernanny audio samples than to clinical audio samples, and if so, we could observe a drop in model performance when evaluating the model on clinical data.

Additionally, for purposes of scream classification, this study does not discriminate between screams evoked by different emotions, a distinction that could be clinically useful. For example, an angry scream produced during a tantrum and a happy scream produced while playing would both be classified simply as “screams” by our model. Although both may be useful as emotional outbursts, clinical researchers may want to focus on a single type of scream, and such study would require review or postprocessing of the scream audio selected by the model.

The Supernanny scream annotations were not validated by a second researcher. Furthermore, Supernanny episodes are 35-40 minutes in length, but the audio recordings most likely to be clinically informative could be days or weeks in length. If the model’s performance on clinical recordings is analogous to its performance on Supernanny, it may produce too many false positives for the raw output to be usable. However, postprocessing may help to reduce the number of false positives and isolate the true episodes of screaming.

### Comparison With Prior Work

#### Technical Work

Earlier works in the area of scream detection have focused on evaluating different audio feature sets and determining which provide the most accurate classification results. Feature sets that have been tested include MFCCs [18], pitch-based features [19], a bag-of-audio-words feature representation [20], autocorrelation features [17], and various hand-constructed feature sets [21]. Studies before 2015 generally used Gaussian mixture models or support vector machines to classify audio data points [18,19,21]. More recent works have tested deep-learning classifiers like deep Boltzmann machines and deep belief networks [22,23]. Additionally, recent studies have tested robustness to noise, using both artificially generated noise and naturally occurring environmental noise [17,23].

Previous studies use a wide range of training data. Due to the scarcity of publicly available audio databases, training data sets generally fall into 1 of 2 categories: “self-compiled” [18-21] or “self-recorded” [17,22,23]. Self-compiled databases use audio samples from sound effect websites, movies, or other sources accessible to researchers. These sound samples may bear little acoustic resemblance to sounds detected “out in the wild.” Databases recorded by the researchers themselves were more realistic but far smaller. These data sets also tend to be narrow in scope, limiting generalizability. For example, Nandwana et al [17] stitched together examples of speech and screaming from 6 male speakers to form 24 continuous recordings, and Zaheer et al [22] recorded 130 scream sounds and 110 “ah” sounds from 60 people in outdoor settings.

The two studies with the most similarities to our work are Nandwana et al [17] and Girin [23]. Nandwana et al [17] show the ability to segment continuous recordings into “scream” and “nonscream” portions, and train and test their model on noisy data. Girin [23] uses a realistic data set with 4 overlapping sound classes and naturally occurring (not artificially added) noise. This study builds on their work by using a larger, less homogenous, and more challenging training data set, and by testing the trained model on audio samples not originating from the training database.

#### Clinical Work

Although scream detection for clinical purposes does not seem to have been explored, we found several sources proposing methods for tantrums and aggression detection. Lefter et al [24] use a fusion of audio and visual streams for automated aggression detection on trains. Although this work has obvious applications in surveillance, a similar methodology could be used to detect aggression or anger in the home.

Current solutions for tantrum detection involve wearable devices combined with parent report [8,25]. The proposed approach for scream detection could be combined with either video-based aggression detection or wearable sensors to improve their accuracy in detecting tantrums or arguments. Another possible method for tantrum detection involves using scream detection to turn a video stream on and off, then allowing parents and caregivers to review the resulting videos and send to clinicians. This parental review process might also alleviate privacy concerns surrounding in-home video collection.

Our proposed method of information extraction may also be used to assist in behavioral management techniques like the “functional behavioral analysis.” This type of clinical assessment has been proven effective in analyzing and designing treatment plans for problem behaviors but can require as many as 40-50 clinician observation sessions per child [26,27]. Video analysis and telecommunication have already been implemented to aid teachers in functional analyses at school with some success [28,29], and the same strategy could be applied using home video. Accurate home video segmentation could provide examples of the problem behavior if it involves or provokes screaming and reduce the need for clinician observation of the child.

The major barriers that remain for using scream detection analysis for routine clinical care relate to identifying conveniently ascertained but also relevant clinical samples that will have generalizability to real-world clinical applications. Screams and verbal outbursts are associated with behavioral disruption and are unpredictable, occurring infrequently but severely in some children while presenting more mildly with increased frequency in other children. The key to effective design of a clinical application will be the ability to ascertain video and audio samples triggered by child screams rather than collecting indeterminant hours of nondisruptive social interaction. The technology, privacy, and accuracy of this approach remains to be explored.

### Conclusions

In this study, we present an approach for detecting screams in home audio recordings with the aim of segmenting those recordings to review clinically relevant portions. The proposed machine-learning approach draws training data from Google’s AudioSet database, uses their associated CNN for feature extraction, and classifies data points with a gradient-boosted tree model. All of these tools are publicly available. The model has achieved an AUC of 0.86 on audio from the training data set, and an AUC of 0.91 on balanced data selected from episodes of the television show Supernanny. Furthermore, it has achieved an AUC of 0.95 and an average precision of 0.42 on the entire unmodified stream of Supernanny audio. These results suggest a possibility that a scream-detecting classifier for clinical audio recordings could be trained using a public database. Additionally, if scream information from audio is used to segment a home video stream, the resulting video clips could be relevant for behavioral disorder diagnosis or management in disorders like autism spectrum disorder or Prader-Willi syndrome. In future work, we plan to obtain and test on clinical data samples to further these hypotheses.

## Abbreviations

AUC: area under the curve
CNN: convolutional neural network
MFCC: mel-frequency cepstral coefficient
ROC: receiver operating characteristic
SNR: signal-to-noise ratio
