# Figure Correction: Detecting Screams From Home Audio Recordings to Identify Tantrums: Exploratory Study Using Transfer Machine Learning

**DOI:** 10.2196/21591

**Published:** 2020-07-08

**Authors:** Rebecca O'Donovan, Emre Sezgin, Sven Bambach, Eric Butter, Simon Lin

**Affiliations:** 1 The Abigail Wexner Research Institute Nationwide Children's Hospital Columbus, OH United States; 2 Department of Psychology Nationwide Children's Hospital Columbus, OH United States

In “Detecting Screams From Home Audio Recordings to Identify Tantrums: Exploratory Study Using Transfer Machine Learning” (JMIR Form Res 2020;4(6):e18279) an error was noticed.

Figure 4 included an incorrect version of the ROC and Precision-Recall curves, which did not reflect the average precision (0.42) and AUC (0.95) reported in the manuscript. Figure 4 has been updated with the correct image and caption.

**Figure 4:**
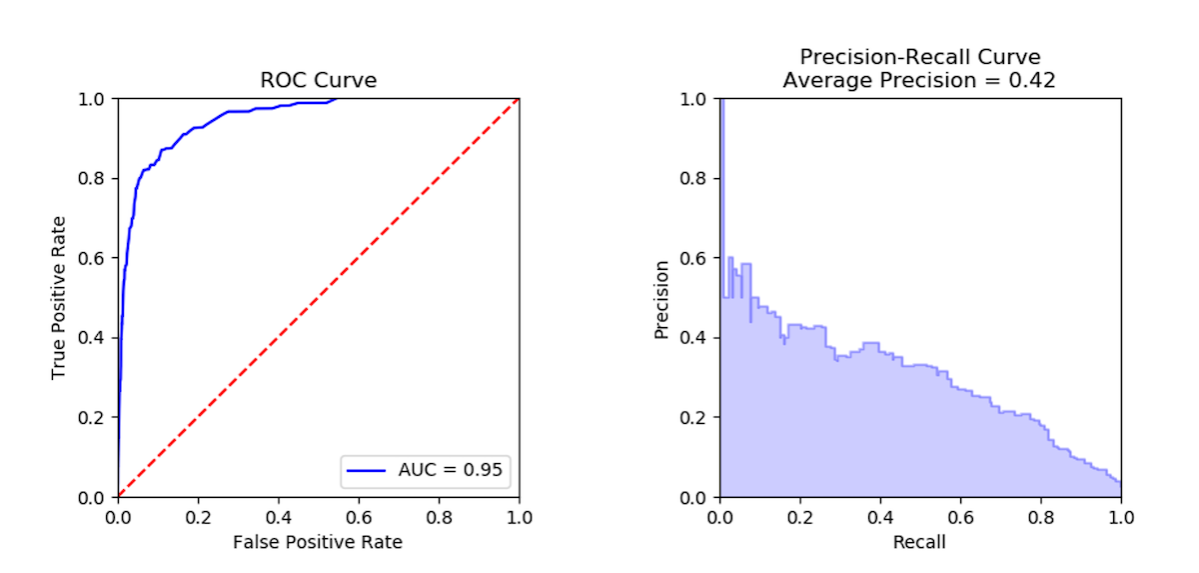
Results on all Supernanny data. AUC: area under the curve; ROC: receiver operating characteristic.

The correction will appear in the online version of the paper on the JMIR website on July 8, 2020, together with the publication of this correction notice. Because this was made after submission to PubMed, PubMed Central, and other full-text repositories, the corrected article has also been resubmitted to those repositories.

